# Expanding the agenda for addressing mistreatment in maternity care: a mapping review and gender analysis

**DOI:** 10.1186/s12978-018-0584-6

**Published:** 2018-08-28

**Authors:** Myra L. Betron, Tracy L. McClair, Sheena Currie, Joya Banerjee

**Affiliations:** 1 USAID’s Maternal and Child Survival Program/Jhpiego, 1776 Massachusetts Avenue, NW Washington DC, 20036 USA; 2 Jhpiego, 1776 Massachusetts Avenue, NW Washington, DC, 20036 USA

**Keywords:** Disrespect and abuse, Mistreatment, Gender, Maternal health, Quality of care, Respectful maternity care (RMC), Gender-based violence

## Abstract

**Background:**

This paper responds to the global call to action for respectful maternity care (RMC) by examining whether and how gender inequalities and unequal power dynamics in the health system undermine quality of care or obstruct women’s capacities to exercise their rights as both users and providers of maternity care.

**Methods:**

We conducted a mapping review of peer-reviewed and gray literature to examine whether gender inequality is a determinant of mistreatment during childbirth. A search for peer-reviewed articles published between January 1995 and September 2017 in PubMed, Embase, SCOPUS, and Web of Science databases, supplemented by an appeal to experts in the field, yielded 127 unique articles. We reviewed these articles using a gender analysis framework that categorizes gender inequalities into four key domains: access to assets, beliefs and perceptions, practices and participation, and institutions, laws, and policies. A total of 37 articles referred to gender inequalities in the four domains and were included in the analysis.

**Results:**

The mapping indicates that there have been important advances in documenting mistreatment at the health facility, but less attention has been paid to addressing the associated structural gender inequalities. The limited evidence available shows that pregnant and laboring women lack information and financial assets, voice, and agency to exercise their rights to RMC. Women who defy traditional feminine stereotypes of chastity and serenity often experience mistreatment by providers as a result. At the same time, mistreatment of women inside and outside of the health facility is normalized and accepted, including by women themselves. As for health care providers, gender discrimination is manifested through degrading working conditions, lack of respect for their abilities, violence and harassment,, lack of mobility in the community, lack of voice within their work setting, and limited training opportunities and professionalization. All of these inequalities erode their ability to deliver high quality care.

**Conclusion:**

While the evidence base is limited, the literature clearly shows that gender inequality—for both clients and providers—contributes to mistreatment and abuse in maternity care. Researchers, advocates, and practitioners need to further investigate and build upon lessons from the broader gender equality, violence prevention, and rights-based health movements to expand the agenda on mistreatment in childbirth and develop effective interventions.

**Electronic supplementary material:**

The online version of this article (10.1186/s12978-018-0584-6) contains supplementary material, which is available to authorized users.

## Plain English summary

Addressing gender inequality is considered a potential strategy for promoting respectful maternity care because mistreatment during childbirth may be a result of low prioritization of women. This review searched for published and unpublished studies that identify inequalities faced by women that contribute to mistreatment during childbirth. The studies selected for review had a range of methods and scope; most examined the gender-based norms and perceptions or practices that lead to mistreatment. However, few documented interventions that address the causes of mistreatment. We concluded that there is still much more research and evaluation to be done to understand and address gender inequality as a driver of mistreatment during childbirth.

## Background

Access to evidence-based, respectful, good quality maternity care is a human right [1]. It is also critical to ending preventable maternal and newborn death in resource-poor settings. There is now a significant body of research on the prevalence of mistreatment of women during maternity care, including physical, sexual and verbal abuse, stigma and discrimination, failure to meet professional standards of care, poor rapport between women and providers, and health system constraints and conditions [2, 3]. Evidence suggests that in countries with high maternal mortality, women are deterred from visiting facilities for maternity care because they fear mistreatment or neglect, based on their own negative experiences and facilities’ poor reputation. As a result, some women prefer to deliver at home with traditional providers who may be more culturally competent or offer more compassionate care [2]. However, home-based births significantly raise the risk of maternal and newborn mortality and morbidity [3].

The White Ribbon Alliance (WRA), which spearheaded the Universal Rights of Childbearing Women in the Respectful Maternity Care Charter, recognizes gender as a factor in respectful maternity care. WRA has stated that because motherhood is specific to women, “issues of gender equity and gender-based violence are also at the core of maternity care, so the notion of safe motherhood must be expanded beyond the prevention of morbidity or mortality to encompass respect for women’s basic human rights” [4]. To ensure gender equity, which is the process of being fair to women and men, health systems must take measures to compensate for historical and social disadvantages that prevent women and men from operating on a level playing field. They must also take action to prevent all forms of violence directed at women based on their biological sex, gender identity, or perceived adherence to culturally-defined expectations of what it means to be a woman.

Population-based research strongly points to the impact of women’s low status on their health, agency, and likelihood of experiencing violence. Data from the most recent Demographic and Health Surveys in Africa and Asia indicate that in many countries the majority of women are not the primary decision-makers for their own health care [5–8]. Likewise, a series of population-based studies in 10 countries found that 30% to 60% of women experience intimate partner or sexual violence, with many women believing their male partners have the right to beat them for a variety of reasons [9]. Gender discrimination and inequality are also emerging as prominent issues for the health workforce. Female health workers regularly face degrading working conditions and reduced compensation as feminized professions such as nursing and midwifery are devalued [10]. Experiences of disrespect, subordination, and gender discrimination were a common finding in the recently released *Midwives Voices, Midwives Realities* report, in which 20% to 30% of respondents said they were treated badly because of discrimination against women and gender inequality [11]. In a survey of 123 countries, women made up 67% of the workforce in the health and social sectors in 2016, compared with 41% of the workforce across all sectors [12].

Some have argued that gender inequality and under-resourced health systems go together—that is, that maternity services receive insufficient investments because they only serve women [13]. However, this link has not fully been researched or analyzed. Moreover, a focus on—or investments in—women’s health is not the same as addressing underlying inequalities in gender norms, attitudes, roles, and behaviors that contribute to the mistreatment of women. These include restrictions on women’s decision-making ability, resources, and mobility; their additional household and caregiving burdens; and the violence they face in various spheres of their lives. Usually, researchers do not frame these issues in terms specific to gender inequality, and practitioners do not address them as factors that could determine the failure or success of programs.

The objectives of this mapping review were to better understand: 1) whether research substantiates the supposition that mistreatment during childbirth is, in part, a byproduct of gender inequality and women’s low status; and 2) to what extent interventions to promote respectful maternity care (RMC) during childbirth address gender inequalities and the poor status of woman as drivers of that mistreatment. Our aim is to explore how current research and interventions articulate the gender dimensions of RMC from both a client and provider perspective.

## Methods

We conducted a mapping review to identify gender-related barriers to RMC experienced by clients, as well as interventions that address these barriers. A mapping review allows for the contextualization of an issue within the broader literature and identification of gaps in the evidence base. Mapping reviews do not exclude items based on study design or involve quality assessment, but still methodically characterize the literature, often with the aim of identifying the need for further research [14, 15].

### Search strategy

We conducted a search for peer-reviewed articles published in English between January 1995 and December 2016 in four databases: PubMed, Embase, SCOPUS, and Web of Science. Key search terms were identified following a preliminary review of the literature. We ran searches in each database using the following seven search terms separately in conjunction with “respectful matern* care”: abuse, gender, disrespect, violence, quality of care, mistreatment, and childbirth. We later repeated our search of the four databases, using the same search terms, to identify articles published between January 2017 and September 2017.

In addition, we solicited articles and gray literature from members of the Global Respectful Maternity Care Council, the Sexual Violence Research Initiative, and the White Ribbon Alliance and from experts on RMC at Jhpiego and the United States Agency for International Development (USAID) Maternal Child Survival Program. This request led us to a seminal paper on the barriers to midwifery care by Filby and colleagues, who identified gender inequality as a key factor underpinning quality of care by midwives: “Midwifery is unique within healthcare, being represented nearly exclusively by women and traversing both domestic and medical domains and cultures” [10]. We reviewed the references in this paper and identified 13 articles on gender as a barrier to midwifery care to include in our review.^1^ Although we did not originally intend to examine gender inequalities affecting midwives and other health care providers and the provision of RMC, the Filby article as well as two others identified through the call for papers warranted further examination into these linkages to quality of care.

### Study screening and selection procedures

**Fig. 1:**
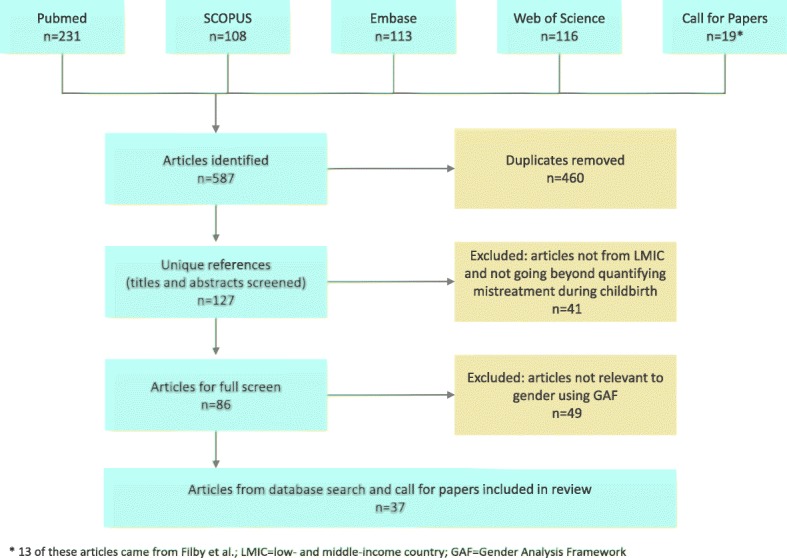
Search and review process

The search strategy yielded 574 articles (Fig. 1). After duplicates were removed, 114 articles remained. Reviewers screened the titles and abstracts of these 114 articles and dropped 41 articles that (1) were not from low- and middle-income countries (LMICs), where the authors and the funder (USAID) work; or that (2) identified, classified, or quantified mistreatment during childbirth without analyzing causes or risk factors or describing strategies to address it. This left 86 articles: 51 from the initial search in December 2016, 22 from the additional search in September 2017, and 13 from the Filby mapping review on barriers to quality midwifery care.

### Data extraction and analysis

At least one author of this paper reviewed the full text of each of the remaining 86 articles to identify and analyze whether it contained information relevant to gender issues or inequalities using the USAID Gender Analysis Framework (GAF) [16]. Gender analysis, as defined by USAID, is an analytic, social science tool that is used to identify, understand, and explain gaps between males and females that exist in households, communities, and countries, and the relevance of gender norms and power relations in a specific context [16]. Such analysis typically involves examining differences in the status of women and men and their differential access to assets, resources, opportunities and services; the influence of gender roles and norms on the division of time between paid employment, unpaid work (including subsistence production and care for family members), and volunteer activities; the influence of gender roles and norms on leadership roles and decision-making; constraints, opportunities, and entry points for narrowing gender gaps and empowering females; and potential differential impacts of development policies and programs on males and females, including unintended or negative consequences.

This framework was originally developed by gender experts and researchers for a manual for integrating gender into reproductive health programs. It is the framework used by the Maternal Child Survival Program, USAID’s flagship program for ending preventable maternal and child death and the sponsor of this study. Using this framework, 49 articles were excluded from the final review because they were not relevant to gender using the GAF. This left 37 articles for final review.

**Fig. 2:**
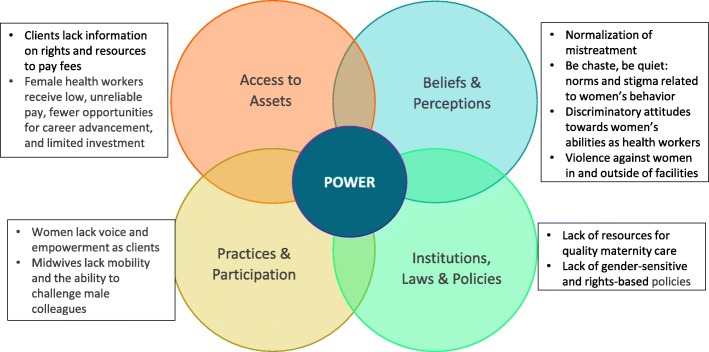
Gender-related drivers of mistreatment during childbirth, organized by USAID Gender Analysis Framework domain

Key findings of each of the final 37 articles were summarized according to the four GAF domains: access to assets; beliefs and perceptions; institutions, laws and policies; and practices and participation (see Fig. 2). A spreadsheet was created to organize the qualitative data extracted from the studies, including types of abuse assessed, geographic focus, study methods, key findings on gender-related issues, gender analysis domains, and subthemes. The lead author then reviewed the summaries to code key findings from each article to one of the GAF domains and tagged each article with a primary theme, e.g., violence, lack of empowerment. Some issues fell into more than one domain and often intersected and compounded each other across domains. All relevant issues identified were included in the paper, but each issue presented in an article was mapped to only one domain.

Although intersectionality was not part of the initial framework for analysis, it emerged as a common theme in the items reviewed. Intersectionality refers to peoples’ various social identities (race, gender, class, age, sexuality, educational status, professional status, single motherhood) in the context of related systems and structures of power [17, 18]. We found that these social identities drive mistreatment during childbirth along with gender-related barriers to RMC and contribute to compounded disadvantage and oppression. We present findings related to intersectionality at the end of the Results.

## Results

The 37 articles included in this review were classified into primary domains, and in many cases, secondary domains. One article was mapped to three domains (see Additional file 1: Table S1). The most common domain was beliefs and perceptions (19 articles), followed by practices and participation (10 articles), access to assets (11 articles), and institutions, laws, and policies (4 articles). Twelve articles cut across various socio-demographic factors for discrimination and thus were categorized under intersectionality. Ten of the articles were global in scope, while 19 reported on 11 countries in Africa, five in Asia, and three in Latin America. Results of the thematic analysis are presented below for each domain and for intersectionality.

### Beliefs and perceptions

This domain focuses on the cultural belief systems or norms about what it means to be a man or woman in a specific society. These beliefs affect men and women’s behavior, dress, participation, and decision-making capacity. They also facilitate or limit men’s and women’s access to education, services, and economic opportunities.

#### Normalization of mistreatment

The literature suggests that many forms of mistreatment during childbirth are normalized so they are not considered a problem; as a result, women have low expectations of care [19–22]. In a global landscape analysis on categories and drivers of abusive maternity care, Bowser and Hill found that women generally accepted the abuse meted out to them because they had never experienced any other type of care; a key informant in a structured group discussion held at Women Deliver said, “They don‘t object or speak out. They accept what they get” [21]. In a cross-sectional study of 173 women in Ethiopia, only 22% of mothers reported experiencing “disrespect and abuse” during childbirth, but that figure climbed to 78% when women were asked about specific types of abuse, such as violations of informed consent, lack of choice of companion, abandonment, and physical harm [19]. Similarly, a qualitative study in Tanzania reported that most women described their facility-based birth as satisfactory despite evidence of discrimination, verbal and physical abuse, abandonment when in need of care, extortion or unofficial fees, and detention in facilities for inability to pay [20].

#### Be chaste, be quiet: Norms and stigma related to women’s behavior

Women who transgress accepted gender norms and defy traditional feminine stereotypes of chastity and serenity often experience mistreatment by providers as a result. In Argentina, Vacaflor describes how gender stereotyping (i.e., making assumptions about women based on beliefs of characteristics or traits associated with gender) drives health personnel’s objectification of women as mere vessels for birthing children, without the capacity to control their own bodies or to understand their experiences [22]. Rather than empowering women with information and choices about their health care, providers “thwarted women’s capacity to act with agency over their pregnancies,” for example, by deciding what position women will deliver in, whether they can have a birth companion, and whether they can deliver vaginally [23]. Participatory action research in the Dominican Republic identified similar values underpinning abuse: women were expected to maintain a “pleasant and even temperament throughout pregnancy”, because they believed that feelings of anger or depression might influence the temperament of the baby [24].

Another common theme is providers’ belief that mistreatment and pain during childbirth are apt punishment for women who engaged in something often viewed as dirty or sinful: sexual intercourse. For example, anthropologists in Mexico documented providers joking and judging women in childbirth by saying “Now you may scream in pain, but nine months ago you were screaming in pleasure.” [25] Providers impose their morals, beliefs, and superstitions on women, often resulting in judgment, blame, and mistreatment during service delivery. For example, in Sierra Leone (and other West African countries) it is widely believed that obstructed labor is caused by infidelity, and some providers insist upon a “confession” prior to providing care [21].

#### Disrespect for women’s abilities as health workers

The literature reveals a lack of confidence in women’s competency at every level of the health system. In a national study of Rwanda’s health employment system that involved written surveys, key informant interviews, and focus groups, female health workers were stereotyped as unwilling to speak up, weak, indecisive and incompetent: “Women are not capable of pulling a tooth.” [26] Discriminatory attitudes toward female managers manifest themselves in stereotypes regarding women’s emotionality, mood swings, tendency to make mistakes, productivity, reliability, organization, vengefulness, mental agility, ability to handle power, weakness, decisiveness, and competence [27].

Gender inequality faced by midwives can be so extreme that it leads to moral distress, burnout, poor retention, and a struggle to provide good quality, respectful care [10, 27]. Mumtaz et al. noted that there is often no career structure for female health workers, particularly in cultures where senior management positions are reserved for men; women may work for decades without receiving a promotion or raise. The authors described that “Time and again respondents expressed their lack of motivation to work hard because there is no appreciation or reward” [28].

Negative attitudes presumably contribute to the lack of women in senior positions in the health care system [10, F. McConville, personal communications, August 22, 2016^2^], although other factors are also at work, including sexual harassment, lack of supportive policies for mothers in workplaces (e.g., breastfeeding rooms or flexible hours), and gender-based discrimination [27, 29] A study on nurse perspectives on the drivers of poor maternal birth outcomes in Nigeria noted that “[t]hough unionized in a country with significant gender inequity, nursing, being a field dominated by females, nurses will never be allowed to rise so high” [30].

#### Violence against women inside and outside of facilities

Findings point to strong parallels and linkages between intimate partner violence and the mistreatment (including outright violence) that women experience during childbirth. Jewkes and Penn-Kekana, leading researchers on violence against women, argued in a commentary for *The Lancet* (2015) that mistreatment during childbirth is a form of violence against women:

“The essential feature of violence against women is that it stems from structural gender inequality, i.e., women’s subordinate position in society as compared to men. This systematically devalues the lives of women and girls and thus enables the inappropriately low allocation of resources to maternity care that is found in many countries. It also disempowers women and enables the use of violence against them” [13].

Earlier qualitative research by Jewkes, Abrahams, and Mvo found that violence by nurses against clients is highly normalized and a method of controlling clients in facilities. Female nurses deployed violence against clients to create social distance and maintain “fantasies of identity and power in their continuous struggle to assert their professional and middle class identity” [31]. Chadwick argues that undervaluing of women leads to acceptance of “obstetric violence”^3^ [32]. A qualitative and quantitative study of 38 primary health care nurses in South Africa also probed the connection between the violence that health workers experience in their own lives and their perpetration of violence toward clients: male nurses shared perspectives that justify violence, such as “women enjoy punishment,” while female nurses shared their own experiences of violence [33].

Providers are vulnerable to violence in the workplace as well as at home, including sexual and physical assault by fellow health workers and community members at large [10, 28]. A 2016 World Health Organization (WHO) report found that 37% of 2470 midwifery personnel surveyed across 93 countries experienced harassment at work, and many described a lack of security and fear of violence [11]. Newman and colleagues found that 39% of health workers surveyed in select facilities in Rwanda experienced some form of abuse in the workplace, including verbal abuse (27%), bullying (16%), sexual harassment (7%), and physical assault (4%); most victims of each form of abuse were female [26]. Of note, Rwandan providers in more gender-equitable health facilities, as measured by perceptions of equitable hiring opportunities and treatment at work for men and women, were less likely to experience violence [26, 28].

### Practices and participation

The norms that influence men and women’s behavior also structure the type of activities they engage in and their roles and responsibilities. This domain captures information on different roles for men and women, when and where their activities occur, and their capacity to participate in decision-making and different types of economic, political, and social activities.

#### Women lack voice and empowerment as clients

Five articles point to women’s lack of voice and agency as clients, even if not explicitly framed as such, and to the roles that men play in negotiating care. In a milestone review, Bowser and Hill found abundant evidence that women generally lack decision-making power in seeking maternity care or delivering in a facility [21]. In Tanzania, McMahon and colleagues found that male partners were more likely than women to assert themselves by voicing concerns, telling a provider to be nicer, or reporting abuse to an oversight body. In contrast, women (as well as some men) reported acquiescing to mistreatment or simply rejecting facilities in favor of home delivery [20]. Additional research in Tanzania found that pregnant women might act submissive for fear of social sanctions [34]. In Kenya, Warren and colleagues found that men paid providers extra money above regular fees for service to ensure quality care for their female partners [35]. Additionally, Warren and colleagues argue that men’s involvement is critical to RMC because of their roles in the household and community [36].

#### Midwives lack mobility and the ability to challenge male colleagues

Three articles found that in conservative settings such as Pakistan and Bangladesh, where there are restrictions on women interacting with men, or on women’s mobility outside the home, there is higher absenteeism, dissatisfaction, poorer job performance, and higher turnover among female health workers [28, 37, 38]. Recognizing that in a culture where women are discouraged from seeing male providers, women’s access to reproductive health care depends on the availability of female health providers, South Asian governments invested in developing a cadre of female health workers “to bring health services within easy reach of largely housebound women” [28]. However, their subsequent efforts to recruit women into the health workforce were stymied by these very gender norms and restrictions. For example, in Pakistan, Mumtaz et al. discovered that female health workers must ask their husband, brother, or mother to accompany them on their duty rounds, severely limiting their functioning and availability. Mumtaz et al. also noted that female health workers in Pakistan are frequently absent or late to work because it is socially unacceptable for women to mix with men on public transport, and few women drive [28]. This can increase the cost, duration, and risk to personal security on the journey to work, to attend trainings, or to visit clients.

Gender and perceived status of providers also factor into power dynamics within the health care team: midwives typically cannot challenge physicians if they disagree with their clinical decisions, which presents a concern for accountable and professional service delivery [10, 39].

### Access to assets

This domain focuses on how gender relations affect access to resources necessary for a person to be a productive member of society. These include both tangible assets, such as land, capital, and tools and intangible assets, such as knowledge, education, and information.

#### Clients lack information on rights and resources to pay fees

Studies in six countries in East and Southern Africa found that women often do not receive information about their care and rights at a health facility [19, 40]. A qualitative study in Tanzania concluded that differences in clients’ and workers’ educational status, as well as lack of knowledge regarding women’s rights, contributes to women’s silence about substandard maternal health services [34].

Two studies found that women’s husbands often broker better care by paying informal fees or bribes. In Tanzania, McMahon and colleagues found that men paid bribes to obtain better care for their partners, while women of a higher social status—especially “women with money”— were prioritized for service [20]. In Kenya, implementation research found that unmarried women were six times more likely to be detained at health facilities than married women at baseline, perhaps because they were less able to pay fees due to lack of financial networks [41]. There may be other unexplored reasons, however, such as lack of information on rights, agency or voice, or other discrimination or exploitation based on unmarried status, as found in research described in the section on intersectionality later in this paper.

#### Female health workers receive low, unreliable pay, fewer opportunities for career advancement, and limited investment

Filby and colleagues found that many midwives across LMICs in Africa and Asia were surviving on wages that failed to meet basic living costs, with salaries paid infrequently, delayed, or not at all [10]. Hurley et al. reported that midwives in Mali relied upon in-kind gifts of food and firewood from the women they served to compensate for the lack of a reliable salary [39]. Mumtaz et al. discovered that low pay caused traditional birth attendants and community health workers in Pakistan to charge clients fees for services that are supposed to be free, and to run private practices outside of work hours for additional income [28]. Worldwide, gender also affects health workers’ access to non-pecuniary rewards, such as housing allowances, vacations, and professional training [27].

Filby and colleagues argue that poor pay—and the resulting financial stress, low self-esteem, and low motivation—is a key driver for the poor quality of care that midwives may provide [10]. Midwives’ low status, which is driven by gender inequality, also accounts for a lack of financial and political commitment to invest in their education, training, regulation, and licensing [10, 42].

Once women become health workers, they face additional challenges. Four articles found that female health workers experience a dearth of opportunities, including a lack of access to pre- and in-service midwifery education [10, 28, 38, 39].

### Institutions, Laws, and policies

This domain focuses on differences in men’s and women’s formal and informal rights and how they are dissimilarly affected by policies and rules governing institutions, including the health system.

#### Lack of resources for quality maternity care

The review found minimal research on and analysis of gaps in institutions, laws, and policies that may contribute to mistreatment during childbirth, beyond the challenge of scarcity of resources. Jewkes and Penn-Kekana argue that limited investment in maternity services stems from the fact that it is a woman’s health issue and thus not perceived as a priority by policymakers [13].

#### Lack of gender-sensitive and rights-based policies

Just five articles addressed the lack of gender-sensitive and rights-based policies. Advocacy organizations like the White Ribbon Alliance and researchers are now framing mistreatment during childbirth as an issue of women’s rights [43]. Freedman and colleagues argue that a strategy to address mistreatment during childbirth needs a strong rights-based framework and advocacy to ensure that women can promote their rights as clients [44].

Small-scale studies of health workers, including some unpublished observations in public and private health facilities in Zambia and Uganda, found a lack of policy responsiveness to the workers’ family responsibilities, evidence of sexual harassment, gender bias in favor of males, and occupational segregation based on gender [27]. Male dominance in the leadership of health governing bodies and institutions has contributed to gender discrimination in the health workforce [27].

### Intersectionality

Although not part of the initial analytical framework, intersectionality emerged as a critical factor that compounds poor treatment and oppression of female clients and midwives. Twelve articles described how discrimination, based on socioeconomic status, education, ethnicity, professional status, and single motherhood, intersects with and aggravates women’s treatment. A review by Mannava and colleagues identified 14 studies—nine set in Africa and five in Asia—that described maternal health care providers working in public and private settings and that demonstrated discrimination toward clients who were poorer, less educated, and rural-dwelling clients, or those belonging to ethnic minorities [45]. A qualitative study in Tanzania reported that rural women of lower socioeconomic status experienced high levels of mistreatment in maternity care [34]. For example, health workers scolded women from rural areas who brought in their babies in old and dirty clothes and abused women who were not able to purchase all the needed maternity care supplies that health workers asked for. Similarly, studies have found that uneducated, rural women of low status in Ghana and poor women in Ethiopia were especially likely to experience abuse [19, 46]. Studies have explored how socioeconomic status leads to “social distance” between providers and clients, which in turn affects the provision of RMC [31, 34, 47]. Hierarchical differences between health workers and clients, based on education and/or class, contributed to female clients’ silence in situations where they knew the health workers were wrong [34].

Ethnic groups that are marginalized in society are often marginalized in clinical encounters as well. Bowser and Hill suggest that rural and indigenous women face higher levels of discrimination based on race, education, traditional beliefs, HIV status, language, age, etc. [21]. For example, Mexican providers forcibly sterilized poor, indigenous women who they believed were promiscuous, ignorant, and non-adherent to doctors’ advice and instructions [48]. Likewise, Whittaker and colleagues [49] found that rural women in Northeast Thailand experienced inequalities related to gender, class, and ethnic relations in health care settings; urban health workers thought poor, uneducated members of ethnic groups were dirty and promiscuous [49].

Not being married or having a partner can also result in discrimination but it may not be the only factor at work. In Mannava’s review of studies in Asia and Latin America, women who were considered social deviants, such as teenage mothers or those undergoing abortion, were also subject to abuse [45]. Amroussia and colleagues highlight single mothers’ experiences of mistreatment in health care facilities in Tunisia and argue that their experiences cannot be explained solely by being single mothers [50]. These women have multiple identities that influence the care they receive, including their gender, poverty, limited education, and raising children without the support of a partner.

Intersectionality may also play a role in the treatment of providers. Studies in Pakistan highlighted how the intersection of class and gender contributed to the disrespect and harassment that lady health volunteers faced from male colleagues and female doctors, who usually belong to a higher class [28, 38].

## Interventions

Few documented RMC interventions address gender, intentionally or comprehensively, as a structural determinant of mistreatment during maternity care, although advocacy at the global and national levels supported by the White Ribbon Alliance has begun to frame the issue as one of women’s rights. A notable exception is the Heshima Project in Kenya, which worked with policymakers to encourage greater focus on mistreatment of women in childbirth, trained and supported providers on RMC, and strengthened linkages between the facility and community for accountability and governance [41]. In particular, Heshima included RMC in the maternal health bill, supporting alternative dispute resolutions between communities and facilities and increasing the visibility of RMC as a rights-based approach for all. Another key Heshima intervention, “caring for the carers,” addressed health system factors that negatively affect health workers and provided opportunities for providers to process work-related stress and pressures [41]. The development and enforcement of a local client charter, including an anonymous client complaint mechanism and feedback surveys, also helped address mistreatment during childbirth [41]. At the community level, sensitization and participatory action planning workshops broke down barriers between providers and clients, engaged male partners, and ultimately contributed to the promotion of RMC [41].

The review identified few other interventions that address gender inequalities, roles, or norms as determinants of mistreatment during childbirth. At the local level, provider trainings focused on values clarification and attitude transformation [33, 41] can help providers develop an understanding of their gender discriminatory attitudes and behaviors in relation to RMC and mistreatment during childbirth. RMC workshops based on curricula such as “Health Workers for Change” engage providers in reflecting on their values and the low status of women and build empathy for client needs [51, 52]. These workshops also look at health worker needs and work with facility leadership and beyond to institute sustainable changes in the health system.

A different approach has been tested in Malawi. A results-based financing scheme rewarded high performing providers in maternal and newborn care with payments. However, no statistically significantly effect was observed on women’s perceptions of care, amenities, or interpersonal relations, and women still reported instances of neglect, disrespect, and verbal abuse. Providers attributed these negative occurrences to an increased workload as more women sought services at facilities supported by the intervention [53].

## Discussion

This mapping review on the role of gender-related factors in mistreatment of women during childbirth found few studies or interventions that take a holistic approach to examining the underlying causes of mistreatment. However, despite the paucity of robust evidence, the literature reviewed does establish the clear relevance of gender inequality—in the form of assets, beliefs and perceptions, roles and norms, and policies and institutions—to the mistreatment of women during childbirth. Further research and programming is clearly warranted.

Beliefs and perceptions about how women should behave, about the normalcy of abuse toward women in health facilities and beyond, and about their abilities as health workers all emerged as strong themes in the literature. Frameworks linking gender and social norms with health outcomes serve as a useful model because they recognize the need to change negative gender norms to improve health [54]. There is growing evidence that gender transformative approaches, that is, strategies that actively seek to change harmful gender norms and power dynamics, can impact health outcomes via client behaviors, for example, by increasing the use of maternal health services, reducing HIV risk behavior, and preventing violence [55]. The RMC field should invest in research to assess similar approaches to reduce mistreatment in childbirth.

Midwives, like clients, face gender discrimination and violence in the work setting, which has negative effects on their wellbeing, morale, and retention. This review did not do a thorough database search on gender inequality in the health workforce and linkages to quality of care, nor did it identify direct links between discrimination against midwives and their mistreatment of laboring women. However, a mapping review by Filby and colleagues makes a strong case that gender discrimination is intertwined with limited investments in midwifery education and training and the slow advancement of midwifery as a profession; thus, they argue that gender discrimination poses a key barrier to good quality midwifery care [10]. On the other hand, in some countries--where female only candidates are mandated and where males and females are accepted--selection and recruitment to midwifery is not a choice but rather dependent on final school examinations, which often results in low motivation and retention. Health systems should test and evaluate structural approaches to addressing the overall devaluation of women, which leads to poor investment in midwifery as a profession, disregard for their skills, harassment and abuse, and overwork.

Gender-based practices and participation were the second most common theme in the review. These are manifested in women’s lack of voice, decision-making power, and mobility, whether as clients or health workers. Evidence-based approaches to empower women as clients through collective action and building social capital, such as participatory action groups for women or care groups, warrant further examination. A meta-analysis reported beneficial effects of women’s empowerment groups on maternal and child mortality [56]. The interventions in the study community gatherings so women could meet with a facilitator for several months to identify and prioritize problems, plan actions, and implement locally feasible strategies. Overall, women in communities with participatory action groups experienced significantly reduced maternal mortality (37%) and neonatal mortality (23%) [56]. Although the empowerment effects of these groups on women have not been well measured, qualitative documentation points to the power of the collective to give voice to women’s needs regarding health services [56]. For health workers, the way forward may lie with in-service and pre-service capacity building efforts. Research is needed to explore interventions that highlight and seek to transform power dynamics within the health workforce, as well as building skills, focusing on communication, leadership, and problem solving.

Women’s limited access to informational and financial assets was the third most common gender issue identified in our mapping review. Women lack knowledge of their rights or choices regarding maternity care and the money to pay formal or informal user fees. Low and irregular compensation of midwives leads to financial stress, low motivation, and low self-esteem. Gender inequality is also described as a root cause of the lack of investment in midwifery as a profession and thus in education, training, and benefits for midwives [10]. These findings call for further exploration of educational and economic interventions. For example, results-based financing, which offers monetary incentives for improved performance, has improved some measures of quality in Afghanistan, Zambia, and Zimbabwe, including the duration of consultations, history-taking, and patient counseling [57, 58]. In Indonesia, incentives and performance-based earnings enabled rural midwives to combine public and private practice, raise incomes, and increase the use of skilled birth attendants^2^. However, results have been mixed, and interventions have not focused on RMC.

Literature describing gender-focused policy, legal or institutional underpinnings, or approaches to mistreatment in childbirth is scarce, but the lack of discourse does not mean laws, policies, and institutions promoting gender equality are irrelevant to RMC. Rather, the gap in the literature may signal an untapped opportunity. Advocacy and rights groups can do more to engage the broader women’s rights and feminist movements on this topic and document their effects. Research from the movement to end violence against women, for example, has found that the greatest advances have been made in countries with the strongest feminist movements [59]. As Freedman and colleagues argue, a strategy to address mistreatment during childbirth needs a strong rights-based framework and advocacy for ensuring women’s voices to promote their rights as clients [44]. As ‘quality, equity and dignity’ for all women emerges as a priority, international organizations, including UN agencies, donors, and professional associations such as FIGO can support countries to act on these priorities and respond to what women want and deserve: quality, accessible, affordable, acceptable respectful maternal and newborn health care [1]. Work is still needed to ensure clinical guidelines and national protocols are framed in gender-sensitive and rights-based perspectives and implemented with strong monitoring frameworks that also include gender specific indicators.

Additional strategies are needed to address the extra layers of inequality dogging the most vulnerable women, including the very poor, the least-educated, and ethnic and racial minorities. The rights of pregnant women in prison as detailed in international law have yet to be realised with evidence of mistreatment and stigmatisation of pregnant women noted in high income countries [60, 61]. This area needs urgent gender-focused policy research and advocacy. Although the literature points to greater discrimination for these groups, we found no interventions that differentiated strategies accordingly. Emerging strategies for disadvantaged or minority groups are mainly found in high-income countries, which were outside of the scope of this review.

In Canada, for example, it is well documented that the marginalization of indigenous people contributes to vast health disparities [62]. The maternal mortality rate of indigenous women is twice that of the general population [63]. Many indigenous mothers shy away from seeking services because they are afraid that if they expose their challenges such as homelessness, unemployment, mental illness, addiction, or violence at home, their babies may be taken away from them. The Kind Faces Sharing Places project funded by Merck for Mothers in 2017, aims to improve quality of care by leveraging indigenous knowledge and research methods with the ultimate goal of improving maternal health [64]. The project applies participatory, client-centered, and culturally sensitive approaches to improve respectful, high quality care through an intersectional lens. Indigenous women have an integral voice in the design, development, and governance of the project.

Another important consideration in the promotion of RMC is the role of men. The literature raises an important question: Are men in a better position to elicit or negotiate respectful care? A companion at birth does have a protective effect against mistreatment and can improve the experience of birth, among other medical benefits; hence having a companion of choice at birth is recommended by WHO [65]. However, further research is needed to assess, first, whether men are more effective in playing the role of advocate and safeguard for women and, second, whether men’s power to advocate for better care may undermine the agency of women giving birth. Does male companionship simply reinforce women’s submissive, secondary roles in patriarchal societies and limit their reproductive autonomy? Or does it lead to better health outcomes and higher quality care? What do women prefer? If they do prefer male engagement, how do we elicit and apply women’s perceptions and rights?

### Strengths and limitations

The scope of the review was limited in several respects. Studies reviewed were restricted to the English language and LMICs, which may have omitted some research from articles in other languages, as well as strategies for disadvantaged groups being tested in high-income countries. However, given that the work of the authors is solely in LMICs and the issues and approaches would likely be markedly distinct due to disparity in resources, we focused the review in LMICs. Moreover, our database search terms also did not include midwives or health workers more broadly, although gender inequalities in these groups emerged as relevant topics among the articles identified in the call for papers through our networks. Importantly, inclusion of articles in the review relied on the authors’ analysis of gender in RMC following the USAID GAF, and only one person thoroughly reviewed each article when assessing whether or not to include it. Finally, as is typical in mapping reviews, studies presented in this paper have varying levels of rigor and were included without assessing their quality or possible bias. Most of the studies were small-scale and/or qualitative in nature, because few population-level quantitative studies have explored this topic. Larger population-based studies would allow for statistical analysis of the associations between measures of gender inequality and women’s empowerment, such as decision-making, autonomy, attitudes towards violence, and experience of mistreatment during childbirth.

## Conclusion

There have been important advances in documenting and reducing mistreatment in health facilities and promoting RMC as a basic human right and standard of care. However, less attention has been paid to the structural and systemic gender inequalities that contribute to poor quality of care. These affect both clients and providers. Pregnant and laboring women lack information, voice, and agency to exercise their rights to RMC, while a predominantly female health workforce suffers from degrading working conditions, discrimination, harassment, and lack of career advancement. Neither a quality-of-care nor a rights-based approach to RMC—alone or together—is sufficient to address the underlying inequalities that contribute to mistreatment. It is essential that we address the gender barriers that lead to mistreatment of female clients and health workers in order to accelerate the elimination of preventable maternal deaths. Researchers, advocates, and practitioners should build upon lessons from the broader gender equality, violence prevention, and rights-based health movements to expand the agenda on mistreatment in childbirth and strengthen current approaches.

## Additional file

Additional file 1: **Table S1.** Mapping Review and Gender Analysis Data xlsx Articles Reviewed and Mapped to Gender Domains. (XLSX 18 kb)

## Abbreviations

GAF: Gender Analysis Framework
LMIC: low- and middle-income country
RMC: respectful maternity care
USAID: United States Agency for International Development
WHO: World Health Organization

## Footnotes

1 In many countries, nurse-midwives are referred to as midwives. In this paper we use the term midwife to include nurse-midwives.

2 F. McConville, personal communications: Homer C, Pascal M, Portela A, McConville F. Systematic Review of Interventions to Overcome Barriers to the Provision of Quality Care by Midwifery Personnel. [PowerPoint slides], August 22, 2016.

3 Obstetric violence is defined in Article 15(13) of Venezuela’s Organic Law on Women’s Right to a Violence-free Life, which was the first to define this form of violence as: the appropriation of a woman’s body and reproductive processes by health personnel, in the form of dehumanizing treatment, abusive medicalization and pathologization of natural processes, involving a woman’s loss of autonomy and of the capacity to freely make her own decisions about her body and her sexuality, which has negative consequences for a woman’s quality of life [31].
